# Pets, Purity and Pollution: Why Conventional Models of Disease Transmission Do Not Work for Pet Rat Owners

**DOI:** 10.3390/ijerph14121526

**Published:** 2017-12-07

**Authors:** Charlotte Robin, Elizabeth Perkins, Francine Watkins, Robert Christley

**Affiliations:** 1 Department of Epidemiology and Population Health, Institute of Infection and Global Health, School of Veterinary Science, University of Liverpool, Leahurst Campus, Neston CH64 7TE, UK; robc@liverpool.ac.uk; 2 NIHR Health Protection Research Unit, University of Liverpool, Ronald Ross Building, Liverpool L69 7BE, UK; lizp@liverpool.ac.uk (E.P.); fwatkins@liverpool.ac.uk (F.W.); 3 Health Services Research Department, Institute of Psychology Health and Society, University of Liverpool, Liverpool L69 3GL, UK; 4 Department of Public Health & Policy, Institute of Psychology Health and Society, University of Liverpool, Liverpool L69 3GL, UK

**Keywords:** emerging zoonotic infection, Seoul hantavirus, human-animal interaction, Mary Douglas, risk, social constructionism

## Abstract

In the United Kingdom, following the emergence of Seoul hantavirus in pet rat owners in 2012, public health authorities tried to communicate the risk of this zoonotic disease, but had limited success. To explore this lack of engagement with health advice, we conducted in-depth, semi-structured interviews with pet rat owners and analysed them using a grounded theory approach. The findings from these interviews suggest that rat owners construct their pets as different from wild rats, and by elevating the rat to the status of a pet, the powerful associations that rats have with dirt and disease are removed. Removing the rat from the contaminated outside world moves their pet rat from being ‘out of place’ to ‘in place’. A concept of ‘bounded purity’ keeps the rat protected within the home, allowing owners to interact with their pet, safe in the knowledge that it is clean and disease-free. Additionally, owners constructed a ‘hierarchy of purity’ for their pets, and it is on this structure of disease and risk that owners base their behaviour, not conventional biomedical models of disease.

## 1. Introduction

In the United Kingdom (UK) in 2012, public health authorities became aware of several cases of acute kidney injury associated with infection from a zoonotic, rodent-borne virus; hantavirus. In the UK, the most common hantavirus is Seoul virus, the predominant hosts of which are brown rats (*Rattus norvegicus*). Rats rarely show signs of disease, but are thought to shed the virus through their urine, feces, and saliva. Humans can become infected through the inhalation of aerosolised rodent excreta [1]. Hantaviruses in the UK were not thought to be of concern until Seoul virus was detected in a patient with acute kidney injury in 2012 [2]. The patient had regular exposure to wild rats (*R. norvegicus*) at their home. Later the same year, Seoul virus was detected in another patient with acute kidney injury [3,4]. This patient had two pet rats (*R. norvegicus*), which were identified as the source of the infection. Subsequently, a national serosurveillance study determined an overall hantavirus seroprevalence of 34% in a sample of 79 pet rat owners, of which the majority (26/27) were seropositive for Seoul virus, and one had a weak positive reaction to Hantaan virus [5], a more severe hantavirus. Since 2011, there have been eight clinical cases of Seoul virus associated with pet rats in the UK [6]. A high Seoul virus prevalence (81%) has also been detected in a sample of 21 pet rats from one breeding colony in the UK [6]; therefore, it is likely that there are more cases of Seoul virus that are currently unreported or undetected.

The public health response to the emergence of Seoul virus was to try and educate rat owners about the potential risks posed by their pet. However, one common misconception about communicating health messages is that knowledge and information drive behaviour [7]. This approach to behaviour change has been widely critiqued, and wider determinants of health behaviour have highlighted the importance of social and structural influences [8]. For example, following the emergence of hantavirus, pet rat owners were advised not to ‘kiss pet rodents or hold them close to (their) face’ [9]; yet messages such as this fail to take into account the way in which pet rat owners live and interact with their rats.

The study of relationships between human and non-human animals and the spaces that animals occupy in human societies has received increasing attention from researchers, propelled by the development of human–animal studies [10]. However, in this field of interest, rats have been somewhat neglected, with few studies focussing on our relationships with this liminal animal [11,12]. Rats are embedded within the Western imagination as ‘vermin’, associated with depravity and filth. They are arguably one of the most vilified animals, “the terror of so many myths and legends” [12]. Rats are also defined as harbingers of disease, notorious for their role in the Great Plague of 1665 and identified as reservoirs for over 60 zoonotic pathogens [13]. This association with disease has been linked to the way in which rats have become “culturally enshrined as one of the most loathed animals on the planet” [14] (p. 86). Yet many people choose to keep these animals as pets, and for these people, none of the powerful associations with dirt, disease, and fear apply. There are numerous meanings attached to this species [15], and these meanings influence the nature of our interactions with them.

In reality, pet and wild rats are the same species; *R. norvegicus*. Regardless of whether it is viewed as a pet or a pest, zoonotic infections such as hantaviruses transcend the boundaries created around constructed categories of animals [14]. Hantaviruses do not respect the boundary between pets and pests; therefore understanding how people construct different categories of animals is essential to the design of health messages for people keeping rats as pets.

Qualitative research methods provide a way of examining how people make sense of their social worlds, and are ideal for studying complex phenomena such as the one presented here [16]. This paper explores the relationship that people have with their pet rats, and how they understand the risk posed by rodent-borne disease. The paper also identifies the implications of this knowledge for health policy makers. Specifically, we were interested in how people view their pet rats as different from wild rats through discourses of purity and pollution. The findings highlight how people’s relationships with their pets and how they perceive different species can influence human health behaviour.

## 2. Materials and Methods

### 2.1. Study Context

This paper is based on a study conducted in the Health Protection Research Unit in Emerging and Zoonotic Infections, which is funded by the National Institute for Health Research. It was a mixed methods study designed to elicit an understanding of disease and risk in human populations in contact with pet and wild rats. The study adopts a symbolic interactionist perspective, starting from the premise that Seoul virus is not a single biological entity, but a collection of meanings produced through identity and social interactions. This approach to examining Seoul virus differs from a biomedical model in which hantaviruses are a universal biological entity. In line with the theory of symbolic interactionism, we used Kathy Charmaz’s approach to grounded theory [17]. This approach enabled an exploration of what individuals construct, and why these constructions evolve [17]. For the purposes of this paper, a subset of in-depth interviews with seven pet rat owners and breeders in England has been analysed. While pet rat owners are identified within the biomedical model as those most at risk of the disease, this study seeks to understand the socially constructed meaning of Seoul virus for them [18].

### 2.2. Study Sample

Qualitative, semi-structured, face-to-face interviews [19] were conducted with seven pet rat owners and breeders between September 2015 and November 2016, including one participant who contracted the Seoul virus in 2016 from her pet rats. Participants were initially identified and contacted via the National Fancy Rat Society (NFRS) list of registered breeders in northwest England [20]. Participants were provided with an information sheet about the study, which informed them that the research was exploring their relationship with and perception of rats and how contact with rats might affect their health and well-being. Initially, three breeders in the study area were contacted via email, and one of these agreed to participate in the study. Subsequently, a combination of social media and snowball sampling were used to recruit further participants, until data saturation was achieved. All subjects gave their informed consent for inclusion before they participated in the study. The study was conducted in accordance with the Declaration of Helsinki, and the protocol was approved by the Veterinary Research Ethics Committee of the University of Liverpool (VREC347).

An interview guide was developed to explore participants’ experiences of rats, health, and illness. The guide covered key areas, which included: exploring the participant’s social identity, how they view rats, how they understand health and illness, and constructs of risk in relation to rodent-borne diseases. Interviews took place at the participants’ home (apart from one, which took place at their work), and lasted between 40–90 min. All interviews were recorded and transcribed *verbatim*.

All of the participants identified as pet rat owners, owning between four and 30 rats each. In addition, two participants were also NFRS-registered breeders. One participant owned pet rats previously, but did not have any at the time of the interview (but had the responsibility for caring for pet rats as part of her job).

### 2.3. Analysis

Initially, interview transcripts were coded line by line, using an open coding approach i.e., codes were not decided *a priori*. This process disassembled the data into discrete parts [21], from the perspective of understanding the meanings that participants attached to rats, health, and well-being, and risks associated with rodent-borne zoonoses. A list of codes was developed, and memos on emerging ideas and possible relationships between codes were kept alongside these initial codes, to be used as a basis for the later development of core themes.

Codes that represented a similar concept were subsequently assembled into conceptual categories [22] with the assistance of the memos. Coding was performed iteratively within and between interview transcripts, using the technique of constant comparative analysis [23]. By interrogating the relationship between these categories, core themes or theoretical codes began to evolve [24].

The constant comparison between data and analysis allowed the development of codes, categories, and theories to be tested across transcripts. Theory was developed further by a review of the literature relevant to the core themes. This process enabled us to develop new theories on how the owners in this study conceptualised their pets and perceived the risks that surrounded them.

## 3. Results

This paper focusses on four themes, which emerged from the interviews with the owners as being relevant to the design of public health messages. Firstly, a ‘tale of two rats’ explores and explains why and how rat owners view their pets as different from wild rats. Secondly, they construct a ‘hierarchy of purity’ within pet rats, which is important in understanding how owners interpret and respond to public health messages. Thirdly, the acquisition of the rat as a pet, its domestication, and the setting in which it is kept created an idea of ‘bounded purity’. The home is seen as a place within which the purity of the rat can be maintained and managed. Finally, we highlight how the way that this particular species is understood by different groups of people has led to two very different responses to the emergence of Seoul virus.

### 3.1. A Tale of Two Rats

Pet rats have a contested status, because the dominant paradigm surrounding this species in society is that they are vermin; the place for rats is outside the home. By keeping rats as pets, people are bringing what is primarily thought of as a pest into the private space of a home. For non-pet rat owners, the rat in the home is out of place. In our interviews, pet rat owners were aware of the deep-rooted associations the animal has with disease:

“*Because society says they are bad and evil and disgusting, dirty things.*”

(P2)

Rat owners talked about themselves and their pets experiencing stigma. Owners felt as though they were unable to talk about their pets with people outside the pet rat community because the rat was not widely considered to be a pet:

“*I think it’s partly, you know, they are an outsider animal. You can say…to people ‘I have a cat’ or ‘I have a budgie’, you don’t get a reaction but rats…they’re still not completely accepted*.”

(P1)

Rat owners recognised the contested nature of their animal as a pet. In making sense of this tension, they created a clear demarcation between domesticated pet rats and wild rats:

“…*in my mind, they are two different things. It’s almost like they share the same name, but aren’t the same thing*.”

(P2)

For pet rat owners, this clear divide between pet and wild rats served a number of purposes. Not only did it go some way towards negating the stigma associated with having pet rats; the act of distancing their pet from its wild equivalent removed its associations with dirt and disease. Their views center on the belief that their pet rat is different from the wild equivalent; once elevated from its “animal status”, the pet rat no longer holds the same powerful associations with dirt and disease that wild rats do.

### 3.2. Hierarchy of Purity

Running alongside this dichotomy between wild and pet rats, pet rat owners also articulated a more complex “hierarchy of purity” among rats. This conceptualisation was used to differentiate pet rats as more or less “pure”, dependent upon their origins. For pet rat owners, the purest types of rat in their hierarchy were those that came from a specialist breeder, and had a genetic background that could be traced. Wild rats were regarded as the least pure, and posed the biggest risk to the pet rat population. In the middle of the hierarchy were pet shop rats and ‘rescue’ rats. Pet shop rats come from an unregulated industry, and are often kept and sold on their own. Keeping a rat on its own is a practice that is not considered acceptable in the pet rat community. There is little control over who buys pet shop rats, and little guidance provided on how to look after them. Rescue rats were seen as ‘impure’ because their breeding could not be traced. Rat owners therefore conceptualised rescue rats as those in need of saving because they were unwanted or rejected, although not necessarily neglected. Interestingly, rescue rats also included rats that had been bought from pet shops. In “rescuing” rats from pet shops, pet rat owners reported saving them from an unsuitable environment and from belonging to someone who would not know how to look after them correctly.

This hierarchy of purity was important for assessing the risk that an incoming rat posed to the pet rat owners’ pre-existing group of rats. Pet rat owners reported preventing ‘impure rats’ coming into contact with their own rats until infection control practices had been implemented. This included taking extra precautions when cleaning out the cages, such as wearing gloves or minimising contact and keeping the rats in quarantine:

“…*like the (pet shop rat), he was quarantined, and all the rescues where I wasn’t sure…if there was any other contact with other rats, then they would have (a) two-week quarantine. Either (at) a friend’s house, who doesn’t have rats, or sometimes upstairs. Which sometimes, it’s not full quarantine because for airborne stuff they would still get it, but at least it means when things transmit through droplets and contact, you are kind of protecting them from that*.”

(P1)

In some cases, owners administered prophylactic antibiotics as a precaution for any infections the rat might have come into contact with outside:

“…*so then you have to treat with antibiotics for two weeks, doxycycline, and then if they do have an infection then that will clear it…so I do take those kinds of precautions because that feels (like) a completely kind of different risk, and more contained as well*...”

(P1)

In all of the interviews with pet rat owners, there was a strong emphasis on the importance of safeguarding the health of the existing rats in the home.

### 3.3. Bounded Purity

The conceptual boundary created by the adoption of the rat as a pet allowed owners to interact with them without considering their potential as a zoonotic disease vector. By controlling the home environment, pet rats were isolated and protected, re-assigning their status from ‘out of place’ to ‘in place’. From the perspective of the owner, the outside world was dirty and contaminated, presenting a risk to the health of the rat. The risks that owners managed were directed at keeping the animal, rather than themselves, safe. Consequently, as pet rats stay in the home, they are only subject to those diseases brought in from outside by humans and other pets:

“*If anything, humans can probably pass things to rats. We are probably more unhygienic than they are, because I leave the house and I come into contact with germs and they’re just sitting in their happy little environment*...”

(P2)

This construction of disease helped to distance the pet rat kept in the home as a harbinger of infection and disease; it was not the rat that was infected, but the environment in which it lived. The critical role of ‘bounded purity’ has been exemplified when this boundary is traversed; when a pet rat transmits hantavirus to the owner, the status of the rat as a pet is removed:

“…*it has definitely changed my relationship with them entirely…I go out to do the rats and that’s them done for the day. I’m not getting them out for five minutes and putting them back and then going back in and out like I was before*.”

(P7)

While in general a pet rat outside the confines of the home becomes ‘out of place’, there were exceptions. Some of the owners attended rat shows. These provided an opportunity to exhibit their animal and receive affirmation of their own identity as a rat owner. These events were often attended by owners with rats from specialised breeders. Competitors attending the shows were required to report any clinical signs that their rats had displayed, as well as any new additions to their rat “family” in the previous two weeks. So, although these events are held outside of the home, the rules under which they operate enabled participants to see them as offering a ‘home-from-home’ environment in which their pet was safe. 

### 3.4. The Divergent Worlds of Pet Rat Owners and Health Officials

Our findings highlight that for pet rat owners, the health of their pets drives infection control practices and biosecurity measures. Their own personal health is not considered in the same way:

“*I think rat people are much more worried about their rats getting sick than they are about themselves getting sick*.”

(P5)

This is important, because the way in which rat owners understand the risk of zoonotic disease is not compatible with conventional scientific models of disease transmission, such as the information provided by Public Health England to inform rat owners about the potential risks of rodent-borne zoonoses [9]. The biomedical model adopted by public health officials as the basis for interventions assumes that the risks rats pose to their owners’ health can be overcome by limiting their contact with the rat. This fails to take into account their interactions with the animal and the way in which pet rat owners view risks associated with their pet:

“…*so yeh I know they have all this advice, but no, I don’t follow it*.”

(P1)

Hantaviruses are asymptomatic in rats; they are invisible diseases. In addition, it is difficult to screen for them in any routine way. Currently, the most accurate method for identifying hantaviruses in rats is a polymerase chain reaction (PCR) test, which identifies the virus DNA in tissue samples (usually kidney), meaning the rat would have to be euthanised. There is a commercial test available that detects hantavirus antibodies in blood samples; an enzyme-linked immunosorbent assay (ELISA). However, an ELISA will only determine whether a rat has been exposed to the virus, not if it is a carrier. This makes it difficult for owners to determine whether their rats have the disease.

Seoul virus complicates the rat owners’ model for disease transmission. The virus has transcended the clear, defined boundary between wild and pet rats, as well as between the ‘contaminated’ outside world and the ‘safe, clean’ world of the home. Furthermore, Seoul virus has been reported in pet rats from specialised breeders, not rescued or pet shop rats, challenging the hierarchy of purity constructed by owners. While the concept of ‘bounded purity’ may work for pet rat owners when clinical signs of disease in the rat are evident and amenable to treatment, Seoul virus does not fulfil these criteria. It is easier to engage with visible, easily recognisable diseases, and to accept them as potential risks; this fits in with rat owners’ disease model.

## 4. Discussion

This paper argues that pet rat owners have their own understanding of the risks presented by Seoul virus, which hinges on how they perceive rats. The way in which Seoul virus is portrayed within the biomedical model of disease does not fit with how they view their pet rats. Owners have knowledge of the disease, how it is transmitted, and are aware of the precautions that they could take to minimise the risk of transmission, but they cannot navigate away from viewing their rat as their pet; a much-loved member of the family and not a potential source of infection.

As a pet, the rat is removed from its wild existence to live a heavily domesticated, bounded life. To pet rat owners, the domestication of the rat has sanitised it, distancing it from the associations with dirt and disease that accompany wild rats. Domestication and construction of an animal as a pet overrides their essential animal characteristics. For pet rat owners, a pet rat is no longer the same as a wild rat; they have almost become two separate species. This construction of the rat as a pet is constantly being challenged by the dominant paradigm of the rat as a pest. Wild rats exemplify the need for defining public and private spaces in discourses of purity, because of the association they have with places on the periphery of human society [25]. As peridomestic rodents, rats occasionally emerge to transgress the boundary between public and private spaces by entering people’s homes [25]. Being able to cross boundaries and defying the natural order gives something ritual power [26]. It could be argued that wild rats possess ritual power; constantly transcending boundaries between the human and animal worlds has manifest in a creature that has a symbolically powerful association with dirt. Wild rats are constantly traversing the boundary between the outside and inside, between the public and private spaces within which humans define what it is to be human. Rats do not stay in their place; they are unbounded. To non-pet rat owners, both wild and pet rats are out of place, regardless of whether they have been invited into private human spaces. Place of encounter has been identified as an important aspect of human–rat relations that shapes how we characterise them [11]. The places where people encounter rats contribute to how the rats are viewed, highlighting how a single animal can have a multiplicity of roles and be associated synchronously with filth and cleanliness.

Arguably, for many people, pet rats are a contradiction in terms, as the archetypal rat is a pest; by keeping rats as pets, this is bringing a pest into the home. This creates disorder and consequently has the potential to subject both the rat and its owner to stigma. In *Purity and Danger*, Douglas proposed an anomaly theory of purity and impurity [26]. The tenet of this theory is that impurity is associated with disorder, of being rejected from a natural order or social structure that societies create to make sense of the world around them. Anything that does not respect this structure becomes polluted, or impure. As Kristeva describes in *Powers of Horror*, abjection is not caused by uncleanliness or disease, but from “what does not respect borders, positions, rules. The in-between, the ambiguous, the composite” [27] (p. 4). Consequently, pollution is never isolated; dirt is not polluting or disgusting in its own right, but can only be understood in relation to a whole system of meaning, a symbolic order that has been created. Through the creation of systems and structures, we create borders or boundaries around certain things or concepts to keep them in their place. “Matter out of place” has the potential to change the way in which it is seen. Douglas argues that for something to become dirty, it needs to be disordered. This implies there is a natural order, a structure by which matter is systematically ordered. Matter becomes dirty when it moves out of this structure and becomes “matter out of place” [26] (p. 36). Our analysis demonstrates that a natural order is too simplistic, and a negotiated social order, which is more fluid, is perhaps a more accurate reflection of how these structures are understood. In the same way that Douglas argues that dirt does not exist independently, concepts such as contamination and impurity are constantly shifting; they can only be understood through a socially produced order. Here, we have defined how pet rat owners construct ‘pure’ and ‘impure’ rats, and how these constructions relate to their understanding of disease risk. Elucidating this understanding has brought to light why pet rat owners appear to be ignoring the risk of Seoul virus, as communicated by public health organisations.

Rat owners’ understanding of the risk of Seoul virus is based on associations between dirt and disease, similar to Douglas’ anomaly theory of pollution. Rats are not inherently dirty; they become dirty because of the environment in which they live. Take the rat out of that environment, elevate it to the status of a pet by placing it in the home environment, and it is no longer perceived to be a risk. Building on this, the analysis in this paper reveals a more complex, negotiated theory of purity and pollution for pet rat owners; a hierarchy of purity.

This hierarchy not only locates the disease threat elsewhere (the outside world), but also creates a diseased “other”; a source of infection far removed from their pets. Sibley argues that the “diseased other has an important role in defining normality and stability” [25] (p. 24). By “othering” wild rats, pet rat owners are reinforcing the status of their pet rats and consequently their pattern of how they understand disease and risk; ‘othering’ wild rats creates stability in pet rat owners’ reality. In groups with a strong sense of identity, which is what we have seen with pet rat owners, a challenge to the hegemony may result in a stricter adherence to community structures. According to Sibley, this is one context in which Douglas’ anomaly theory is reflected in practice: “[In] closed, tightly-knit communities with something approaching a conscience collective, it may be that adherence to the rules is more likely in times of crisis, when the identity of the community is threatened” [25] (p. 38). The emergence of Seoul virus within the pet rat community has threatened their symbolic structure, their hierarchy of purity. Arguably, this may have acted to reinforce their constructions of purity and the “othering” of wild rats; the emergence of Seoul virus has made these structures more fixed and impenetrable, making communication of the risks associated with the disease more problematic.

When communicating risk to pet rat owners, public health professionals have been basing their advice on conventional, biomedical models of disease transmission. The biomedical model of disease has been the dominant paradigm in medicine and health research over the last century [28]. This biomedical model assumes a causal relationship between disease and illness, and that all disease will eventually result in illness. In this model, health is defined as the absence of disease, and the removal of the disease will result in a return to health. Our analysis has demonstrated that these conventional models of disease do not work for pet rat owners. There is dissonance between the biomedical model of health and illness, which focusses on physical and biological aspects of diseases and how the rat community understands Seoul virus as an illness. So rather than ignoring advice, rat owners are unable to act upon it, because of their beliefs and conventions surrounding their pet. Additionally, a biomedical model of disease is problematic for Seoul virus, as not only is it asymptomatic in rats, but exposure to the pathogen in humans does not always equate to a diagnosis. This has been demonstrated by Public Health England, where their seroprevalence study indicated one third of pet rat owners tested were seropositive to the virus; however, not all of these individuals actually experienced the disease by becoming ill [5].

Public health professionals communicated health messages to pet rat owners based on the biomedical model and their own interpretations of risk. This led to them communicating health messages based on the premise that exposure to a rat equates to disease and consequently illness. It has become apparent from our interviews with pet rat owners that they not only have a different understanding of disease models, but they have also interpreted the risk of Seoul virus differently, and these constructions of disease and risk are based on the hierarchy of purity they have developed around their pet rats.

Seoul virus is rejected by the pet rat community because it is ambiguous, composite, the in-between [27]. It is abjection; it does not fit in with their understanding of disease and illness; therefore, it is an anomaly. Rather than responding negatively to this anomaly, by ignoring the risk it poses, owners have responded more positively by creating a new reality in which Seoul virus has a place: a ‘hierarchy of purity’. This example of how people react when confronted with anomalies in their lives provides another illustration of Douglas’ theory being reflected in practice: “…life does not always conform to our most simple categories. There are several ways of treating anomalies. Negatively, we can ignore, just not perceive them, or perceiving them, we can condemn them. Positively, we can deliberately confront the anomaly, and try to create a new pattern of reality in which it has a place” [26] (p. 39). In pet rat owners’ reality, even following instances of the emergence of Seoul virus within pet rats, the virus is a risk associated with wild rats, or any rat that might have had contact with the outside world. In these situations, owners demonstrate an awareness of the risk, which is translated into action to reduce it. They do follow the “good, simple hygiene rules” communicated by public health organisations such as quarantine, hand hygiene, and taking extra care when cleaning out cages, although arguably with their rats’ health as the priority rather than their own. When Seoul virus is reified, when it becomes real rather than an ‘invisible’ threat, the damage caused is more than just the physical effects of the disease. It is not only harmful to physical health, but it also alters the intimate bond between owner and pet, which is a potentially irreversible shift that changes the status of the rats so that they are no longer seen as a pet. When this bond between owner and pet is so important, and the animal plays such a vital role in their social identity, it is understandable why it has been so difficult for owners to conceptualise the risk of their pets transmitting Seoul virus. Rat owners are limited in their ability to determine whether or not their rats carry the virus, and are therefore more likely to operate on the basis that they are not infected, rather than the reverse.

## 5. Conclusions

The emergence of Seoul virus in the pet rat population has highlighted the importance of understanding how people construct disease and risk, particularly when the owner–pet bond is entangled within the route of transmission. Public health organisations communicated health messages to this at-risk group based on conventional biomedical models of disease. For public health organisations, the rat was a common source of infection across all at-risk groups, resulting in the simple message: change how you interact with this potentially infected animal. For pet rat owners, the elevation of the rat to the status of a pet means it sheds all previous associations with dirt and disease. The rat becomes a completely different species. From the public health perspective, rat owners appear to be ignoring the risks presented by Seoul virus. The analysis presented here suggests that these risks identified by public health organisations are not recognised or shared by pet rat owners.

Through a lens of purity and pollution, we have examined how pet rat owners have created a hierarchy of purity for their pets. They believe in this hierarchy rather than the biomedical model, which frames their pet as a disease risk. For owners, rats are not inherently dirty; it is the environment in which they live that contaminates them. Remove the rat from the outside environment, bring it into the home, and the rat becomes clean and therefore safe. In our analysis, the concept of the home has become stretched to include the pet rat so it is no longer out of place; owners have created a new concept of ‘home’ in which the pet rat is included and welcome. In this context, the rat is no longer ‘matter out of place’, because place matters. To change the behaviour of pet rat owners, public health messages must be based on an understanding of how rats are perceived as pets and how owners interact and care for their pets in everyday life. Small changes, such as developing public health messages specifically for pet rat owners, or shifting the focus of messages so that the health of the rat is also taken into account may be more successful at engaging with this population.
